# Inhibition of the MRSA Biofilm Formation and Skin Antineoplastic Activity of Ethyl Acetate Roots and Aerial Parts Extracts from *Geum urbanum* L.

**DOI:** 10.3390/antibiotics14070627

**Published:** 2025-06-20

**Authors:** Lyudmila Dimitrova, Maya M. Zaharieva, Lilia Tserovska, Milena Popova, Vassya Bankova, Hristo Najdenski

**Affiliations:** 1 Department of Infectious Microbiology, The Stephan Angeloff Institute of Microbiology, Bulgarian Academy of Sciences, Acad. G. Bonchev Str. Bl. 26, 1113 Sofia, Bulgaria; zaharieva26@yahoo.com (M.M.Z.); hnajdenski@abv.bg (H.N.); 2 Department of Biology, Medical Genetics and Microbiology, Medical Faculty, Sofia University “St. Kliment Ohridski”, 1 Koziak Str., 1407 Sofia, Bulgaria; l_tserovska@abv.bg; 3 Laboratory Chemistry of Natural Products, Institute of Organic Chemistry with Centre of Phytochemistry, Bulgarian Academy of Sciences, Acad. G. Bonchev Str. Bl. 9, 1113 Sofia, Bulgaria; popova@orgchm.bas.bg (M.P.); bankova@orgchm.bas.bg (V.B.)

**Keywords:** *Geum urbanum* L., MRSA biofilms, gene expression, cytotoxicity and skin neoplasms, skin irritation

## Abstract

**Background:** The opportunistic pathogen *Staphylococcus aureus* causes skin and soft tissue infections that are associated with biofilm formation, and in immunocompromised patients can progress to surgical site infections, pneumonia, bacteremia, sepsis, and even death. Most antibiotics actively damage living, dividing cells on the surface of the biofilm, where there is a high concentration of nutrients and oxygen, while in the depths, where these factors are scarce, slowly growing cells remain. **Objectives:** The aim of our study was to evaluate the antibiofilm potential of ethyl acetate roots (EtOAcR) and aerial parts (EtOAcAP) extracts from the perennial Bulgarian plant *Geum urbanum* L. against methicillin-resistant *S. aureus* (MRSA) NBIMCC 8327. **Methods**: The effects of both extracts on the expression of biofilm-related genes, *ica*A and *ica*D, were investigated. The cytotoxicity of EtOAcR and EtOAcAP on A-375 (human melanoma), A-431 (epidermoid skin cancer) and HaCaT (normal keratinocytes) cell lines, and the induction of apoptosis were determined. Finally, the in vivo skin irritation potential of the most active extract was studied. **Results**: Both tested extracts inhibited biofilm formation at concentrations that did not affect bacterial growth. Interestingly, the expression of *ica*A and *ica*D was upregulated, although the biofilm development was inhibited 72.4–90.5% by EtOAcAP and 18.9–20.4% by EtOAcR at sub-MICs. EtOAcAP extract showed a more favorable cytotoxic profile on non-tumorigenic cells and stronger antineoplastic activity (IC_50_ = 6.7–14.68 µg/mL) as compared to EtOAcR extract (IC_50_ = 8.73–23.67 µg/mL). Therefore, a skin irritation test was performed with the EtOAcAP extract at ten-times higher concentrations than the minimum inhibitory one, and, resultantly, the primary irritation index was equal to zero (no skin irritation observed). **Conclusions**: The EtOAcAP extract was proven to be an effective antistaphylococcal agent with favorable skin tolerance. The extract showed strong antineoplastic activity and antibiofilm effect at sub-MICs, which outlines new prospects for its development as a natural product for specific skin applications in medical practice.

## 1. Introduction

A significant global health concern of the 21st century is antimicrobial resistance (AMR). In 2021, it was reported that of the 4.71 million deaths associated with AMR, 1.14 million were due to bacterial AMR. The number of deaths due to AMR is expected to reach 1.91 million and the AMR-related deaths are expected to reach 8.22 million worldwide by 2050. It has been reported that from 2025 to 2050, the number of deaths due to AMR is projected to reach 39.1 million, and that of AMR-associated deaths is expected to reach 169 million [1].

*Staphylococcus aureus* is one of the most common opportunistic pathogens causing skin infections worldwide. It is known that the toxins Panton–Valentine leucocidin (PVL), exfoliatins (ETs), enterotoxins, and toxic shock syndrome toxin 1 (TSST-1) can cause cutaneous clinical manifestations. From a medical point of view, methicillin-resistant *S. aureus* (MRSA) is a major problem, because it complicates therapy [2]. In nosocomial infections and immunocompromised patients, infections can often progress to pneumonia, bacteremia, surgical site infections, sepsis, and death [3]. The phenomenon of biofilm formation on implants and tissues is often observed, which complicates antibiotic treatment [4]. People with diabetes mellitus are at risk, especially those with decubitus ulcers, which represent an open door for infection [5]. The association between *S. aureus* and the development of squamous cell skin cancer has been shown, explained by the increased expression of human β-defensin-2. It is possible to contribute to the development of breast cancer, bladder cancer, and colon cancer [6]. Melanoma patients are particularly susceptible to MRSA bloodstream infection and die more often than non-cancer patients, due to frequent hospitalizations, chemotherapy, surgical interventions, and the use of broad-spectrum antibiotics [7]. A comparative analysis from 1990 to 2021 shows an increase in deaths associated with and caused by MRSA (the number of associated deaths increased from 261,000 to 550,000, and the number of attributable deaths rose from 57,200 to 130,000) [1]. According to Word Health Organization (WHO) report for 2024, the median resistance percentage to methicillin in bloodstream infections caused by *S. aureus* was 32% in 2021, compared with 13.4% in 2016 [8]. A recent study using meta-analysis demonstrated that mortality in patients with MRSA bacteremia was higher compared to that caused by methicillin-susceptible *S. aureus* [9]. This is why it is extremely important to understand the mechanism of staphylococcal biofilm formation and its subsequent complications if treated incorrectly.

The presence of extracellular polymeric substances (EPS) in the MRSA biofilm, such as poly-N-acetylglucosamine surface polysaccharide (PNAG) and extracellular DNA (eDNA), helps bacteria resist environmental stress and antimicrobial agents by blocking their penetration [10]. The formation of the biofilm occurs in four stages: (1) microbial cell adhesion, (2) aggregation, (3) maturation (bacterial proliferation), and (4) cell dispersion. PNAG, adhesins, teichoic acids, the hydrophobicity of bacterial cell surfaces, and the synthesis of eDNA are some of the mechanisms that lead to attachment [10,11]. In the last stage, phenol-soluble modulins (PSMs) and proteases degrade the biofilm matrix, which enables bacterial cells to disperse from the biofilm and colonize new niches (cell detachment) [12]. On the surface of the biofilm, where there is an abundance of nutrients and oxygen, there are living, actively dividing bacterial cells. Deep within the biofilm, where these elements are less abundant, there are slow-growing and dying cells. Most chemotherapeutics and antibiotics damage actively dividing cells but do not completely destroy bacterial biofilms. This is why most staphylococcal infections persist and recur. The ability of bacteria to adhere to surfaces, their antibiotic resistance, their ability to transfer DNA, and other factors are part of the biofilm formation process, which is controlled by the expression of specific genes [13]. The intercellular adhesion genes *ica*A, *ica*B, *ica*C, and *ica*D, which are encoded within the intercellular adhesin IcaADBC locus (ica operon), regulate the cell-to-cell adhesion process and biofilm initiation—the biosynthesis of polysaccharide intercellular adhesion (PIA), consisting of β-1,6 N-acetyl glucosamine [14,15]. Of the four genes mentioned above, the *ica*A and *ica*D genes play a central role in biofilm formation [16] due to their co-expression of N-acetylglucosaminyltransferase activity, which results in the formation of 20-residue oligomers [17,18].

The perennial herb *Geum urbanum* L., known as St. Benedict herb, is widespread throughout Europe, including the Middle East and Bulgaria, where it grows between 200 and 1600 m above sea level [19,20]. It is found on the market as a commercial product in the form of a dried herb (roots or aerial parts). It is known that tinctures and decoctions of it were applied in Ukrainian and Russian traditional medicine, as the plant has sedative, anti-inflammatory, antiemetic, analgesic, hemostatic, antiseptic, anthelmintic, and antimalarial effects [21,22,23]. In Bulgarian folk medicine, it is used to treat (1) gastrointestinal tract infections such as dysentery and catarrh of the stomach and intestines, accompanied by high fever; (2) diseases of the liver and biliary tract, indigestion, vomiting and intestinal colic; and (3) prolapse of the uterus and rectum [24]. The properties of different extracts from *G. urbanum* have been studied: antimicrobial [25,26], including the inhibition of the quorum sensing system in *Pseudomonas aeruginosa* [27,28], radical-scavenging [26,28], anti-diabetic [28,29,30], and cytotoxic and antiviral [31,32,33,34,35] activities.

In our previous work, the antimicrobial activity against Gram-positive bacteria of ethyl acetate extracts of roots (EtOAcR) and aerial parts (EtOAcAP) was proven. The EtOAcAP showed minimal inhibitory concentration (MIC) 39 µg/mL and EtOAcR 313 µg/mL against MRSA [26]. This study aimed to evaluate the biofilm inhibitory potential of these two extracts at sub-MIC (^1^/_2_MIC, ^1^/_4_MIC and ^1^/_8_MIC) and to examine the expression of the intercellular adhesion *ica*A and *ica*D genes by real-time PCR. In parallel, their therapeutic properties, such as antineoplastic agents, including the induction of apoptosis, have been investigated. Finally, a skin irritation test was performed to assess the safety of the most active extract regarding its potential development as an herbal product with anti-inflammatory and antiseptic activity for the adjuvant skin therapy of malignant diseases accompanied by staphylococcal infections.

## 2. Results

### 2.1. Evaluation of MRSA to Produce Slime (Congo Red Agar Plate Test)

**Figure 1:**
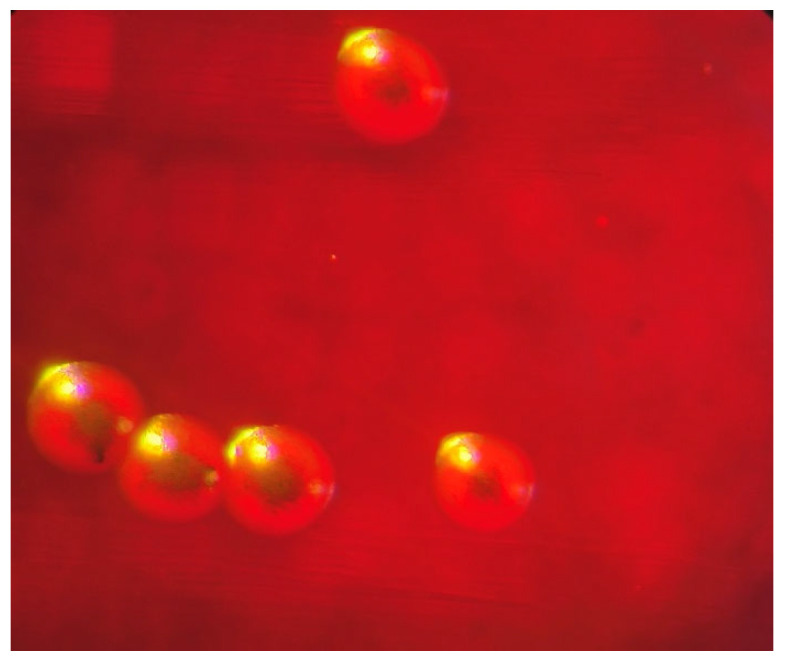
Morphology of MRSA colonies under 25× microscope loupe.

To ensure that the reference strain MRSA would form a biofilm, the ability to produce slime was assessed on a Congo red agar (CRA) plate (Figure 1).

MRSA NBIMCC (National Bank for Industrial Microorganisms and Cell Cultures) 8327 formed black colonies, which under a microscope had a white periphery with a black center.

### 2.2. Evaluation of MRSA Biofilm Formation Capability

**Figure 2:**
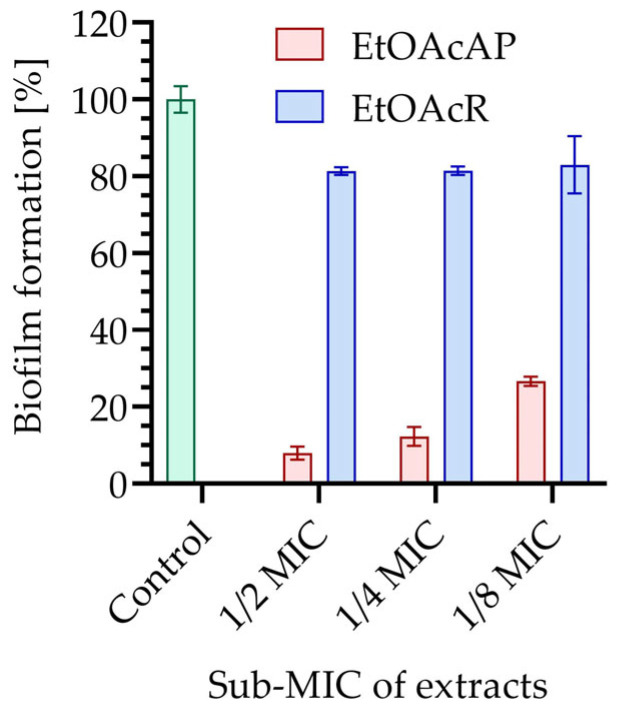
Determination of MRSA biofilm formation capability.

The ability of MRSA to form biofilm without EtOAcR and EtOAcAP extracts (untreated control), and after treatment with them at sub-MICs (MIC of EtOAcAP was 39 µg/mL and MIC of EtOAcR was 313 µg/mL [26]) was determined (Figure 2).

Three sub-MICs of EtOAcR (^1^/_2_MIC: 156.25, ^1^/_4_MIC: 78.12 and ^1^/_8_MIC: 39.06 µg/mL) and EtOAcAP (^1^/_2_MIC: 19.53, ^1^/_4_MIC: 9.77 and ^1^/_8_MIC: 4.88 µg/mL) extracts were selected. At ½MIC of EtOAcAP extract, biofilm formation was inhibited by 90.5%. Even at ^1^/_8_MIC, the same was suppressed by 72.4%. The EtOAcR extract inhibited biofilm formation between 18.9 and 20.4% at the test-subMICs. The statistical significance is presented in Supplementary Table S1.

### 2.3. Gene Expression

**Figure 3:**
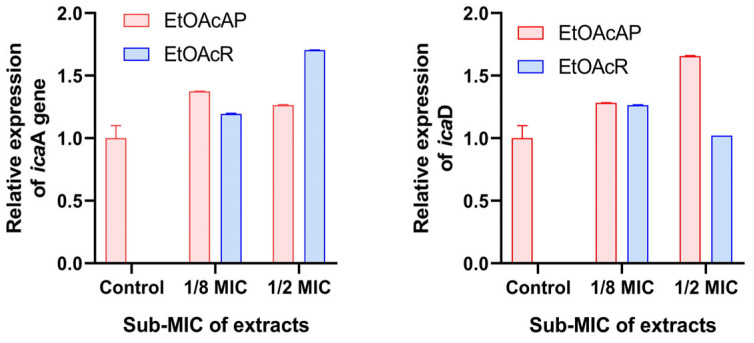
Relative expression of *ica*A (**left**) and *ica*D (**right**) genes (2^−ΔΔCt^) in MRSA exposed to ½ and ^1^/_8_ MICs of EtOAcAP and EtOAcR extracts.

Upon overnight exposure to EtOAcAP and EtOAcR at ^1^/_8_MIC and ½MIC, the expression of the *ica*A and *ica*D genes increased compared to the untreated control. The gene expression was compared to the *rho* housekeeping gene (Figure 3).

The results showed that when the expression of *ica*D increases, the expression of *ica*A decreases, and vice versa, when the expression of *ica*A increases, that of *ica*D decreases. At a concentration of ½MIC EtOAcR (156 µg/mL), the expression of *ica*D gene was equal to that of the untreated control. The statistical significance is presented in Supplementary Tables S2 and S3.

### 2.4. Cytotoxicity and Antiproliferative Activity

**Figure 4:**
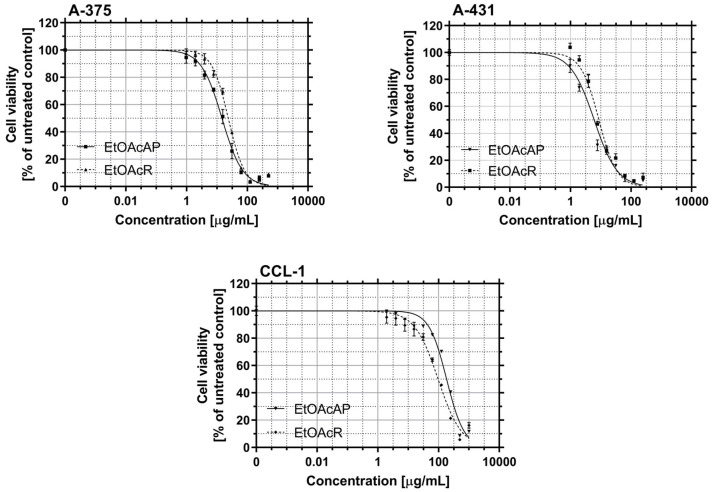
Antiproliferative activity of EtOAcAP and EtOAcR extracts from *G. urbanum* on two tumorigenic (A-375 and A-431) cell lines and cytotoxic activity on one non-tumorigenic (CCL-1) cell line.

The cytotoxic effect of EtOAcAP and EtOAcR extracts from *G. urbanum* on the CCL-1 cell line and their antiproliferative activity on A-375 and A-431 cell lines were evaluated (Figure 4). The IC_50_ and the selectivity index (SI) versus normal fibroblasts are presented in Table 1.

The EtOAcAP extract showed a two times lower cytotoxicity (IC_50_ = 184.3 µg/mL) compared to the EtOAcR extract (IC_50_ = 86.84 µg/mL). The two tested extracts possess high antiproliferative activities. The EtOAcAP extract showed a higher effect against both cell lines (A-375, IC_50_ = 14.68 µg/mL and A-431, IC_50_ = 6.07 µg/mL), as well as high SI. It also exhibited high antibiofilm activity, inhibiting staphylococcal biofilm by 72.4–90.5% at ^1^/_8_MIC-½MIC (Figure 2). Therefore, our research continued by examining only the EtOAcAP extract.

**Figure 5:**
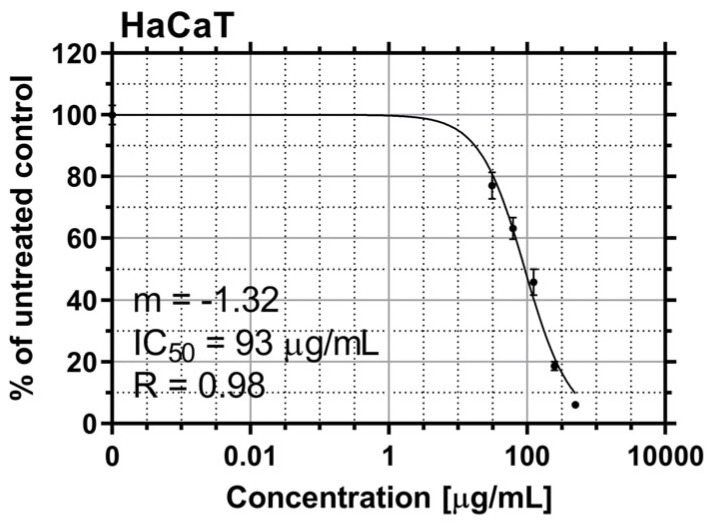
In vitro cytotoxicity of EtOAcAP extract from *G. urbanum* on human keratinocytes (HaCaT cell line).

The cytotoxic effect of EtOAcAP extract was also examined on non-tumorigenic cell lines, human keratinocytes HaCaT (Figure 5), which are a representative comparison to the tumorigenic cell lines.

The average inhibitory concentrations of the EtOAcAP extract in the HaCaT cell line were 6 to 15 times higher than those determined for tumorigenic A-375 and A-431 cells, respectively. Based on these values, SI for the two tumor cell lines were calculated as follows: HaCaT/A-375, SI = 6.33 and HaCaT/A-431, SI = 15.32. The EtOAcAP extract was characterized by a higher SI when applied to the A-431 cell line, compared to A-375 cells.

### 2.5. Assessment of Clonogenicity

**Figure 6:**
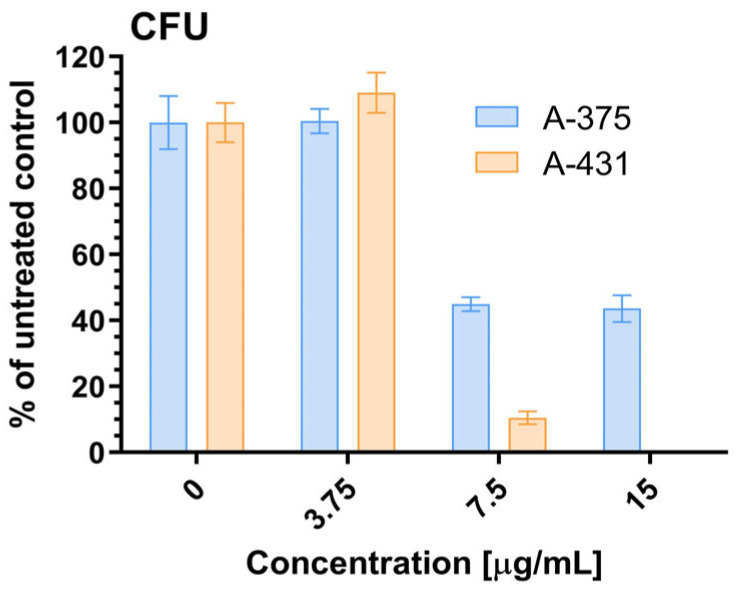
Anticlonogenic effect of EtOAcAP extract from *G. urbanum*.

The effect of the EtOAcAP extract on the clonogenicity of the two tumorigenic cell lines was investigated (Figure 6).

The clonogenicity of A-431 cells was completely inhibited after treatment with 15 µg/mL EtOAcAP, and it was suppressed by over 90% after exposure to a concentration of 7.5 µg/mL. The same two concentrations inhibited the clonogenicity of A-375 cells by approximately 55%. The lowest applied concentration of 3.75 µg/mL had no inhibitory effect on the clonogenicity of both tumor cell lines. The statistical significance is presented in Supplementary Table S4.

### 2.6. Induction of Apoptosis—Caspase 3 Activity

**Figure 7:**
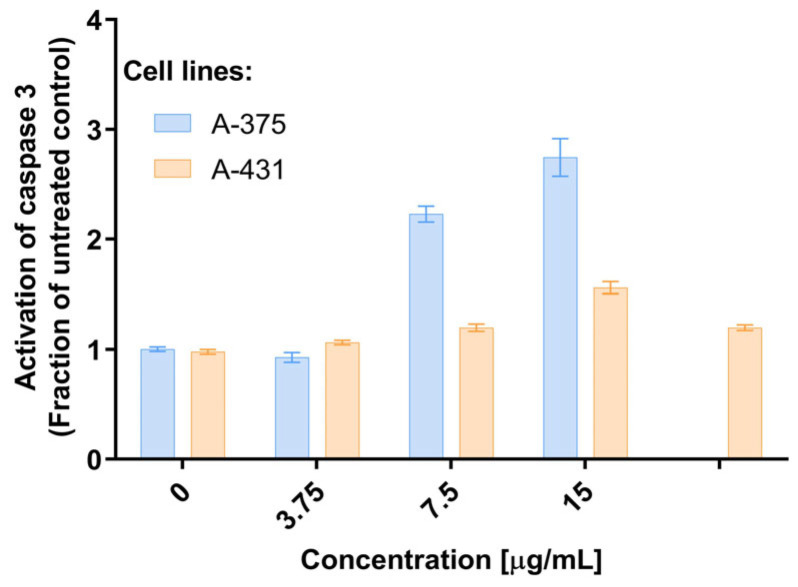
Activation of caspase 3 after treatment of two tumorigenic cell lines (A-375 and A-431) with EtOAcAP extract from *G. urbanum*.

The potential of the EtOAcAP extract to induce apoptosis was investigated by measuring the activity of executioner caspase 3 (Figure 7).

After incubation with the tested extract, there was a concentration-dependent induction of the enzyme activity. After 48 h, it was more pronounced in the A-375 cell line, which is less sensitive than A-431. The statistical significance is presented in Supplementary Table S5.

### 2.7. Induction of Apoptosis—Hoechst Staining

**Figure 8:**
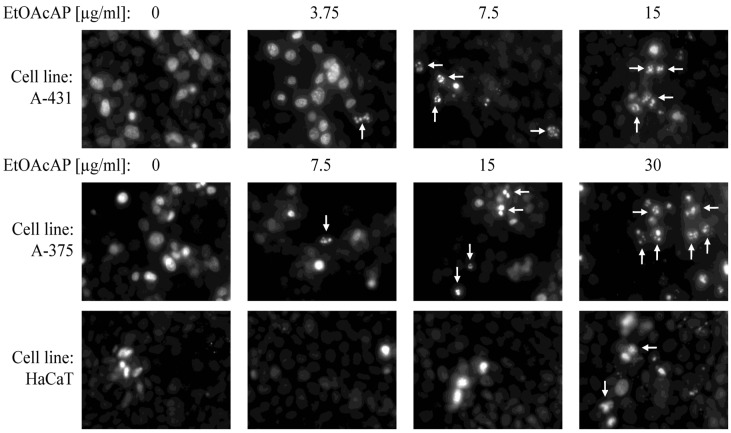
DNA fragmentation after treatment of two tumorigenic cell lines (A-375 and A-431) with EtOAcAP extract from *G. urbanum*—Hoechst staisning. Legend: Arrows indicate DNA fragmentation.

The induction of apoptosis by staining with the Hoechst fluorescent dye by visualizing DNA fragmentation in apoptotic cells was also demonstrated (Figure 8).

Even at a concentration of 3.75 µg/mL, DNA fragmentation was observed, which was concentration dependent. Compared to both tumorigenic cell lines, cellular DNA fragmentation in the HaCaT cells was demonstrated only at 30 μg/mL.

### 2.8. Assessment of Skin Irritation Potential

**Figure 9:**
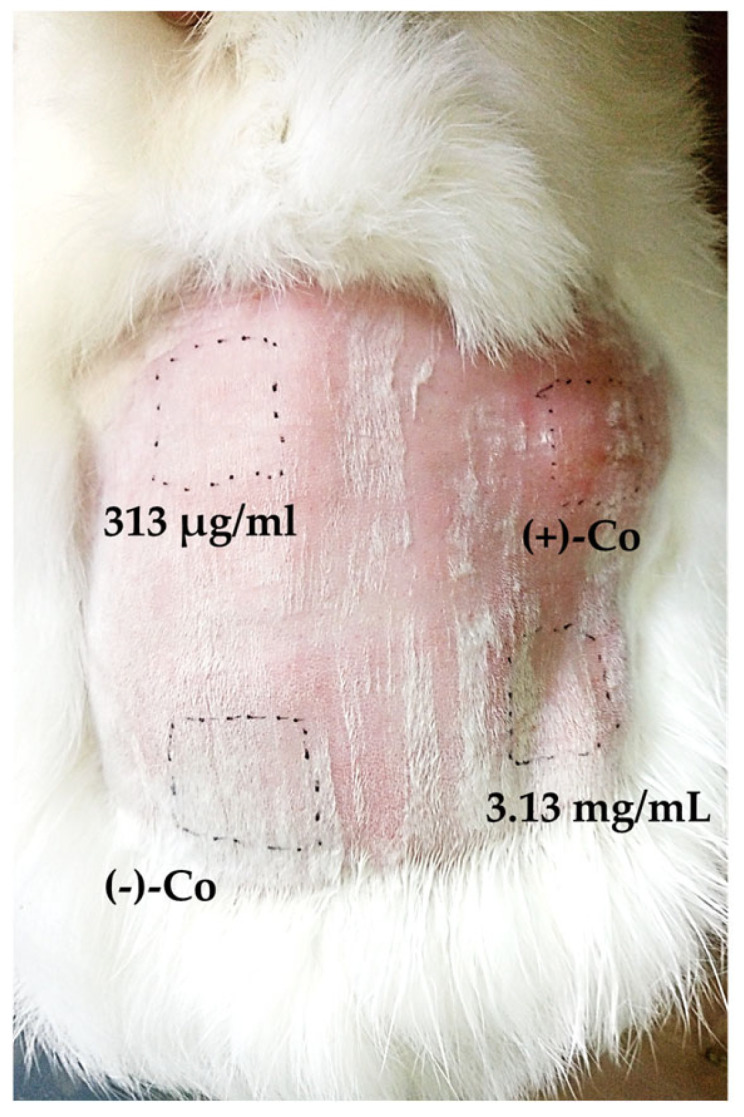
Skin irritation test of EtOAcAP extract at 313 µg/mL and 3.13 mg/mL concentrations at the 72nd hour. Legend: (−)-Co—USP water and (+)-Co—10% SDS.

A skin irritation test was performed to determine the potential of the extract to cause skin irritation at two concentrations—previously determined MIC [26] and 10 × MIC (Figure 9).

The dermal safety of the EtOAcAP extract was evaluated at 24, 48, and 72 h after a 4 h exposure period onto rabbit skin. Figure 9 presents the result after 72 h of exposure as represented by the lack of skin irritation after exposure to the extract. At both tested concentrations (MIC = 313 µg/mL and 10 × MIC = 3.13 mg/mL), the primary irritation score (PIS) and the primary irritation index (PII) were calculated (PIS and PII were equal to zero). In comparison, PIS and PII of the positive control (10% SDS) were equal to 3 (moderate erythema and edema). Since a single exposure to the extract did not cause skin irritation, the cumulative irritation index (CII) was not calculated.

## 3. Discussion

In the present study, the potential of ethyl acetate extracts from roots and aerial parts of the Bulgarian plant *G. urbanum* L. to inhibit MRSA biofilm formation and the expression of the intercellular adhesion *ica*A and *ica*D genes at sub-MICs was investigated. Since patients with skin cancer may have a staphylococcal infection, it was decided to investigate and their preventive and antineoplastic effects. A skin irritation test was conducted to ensure that the most active extract against MRSA with a favorable cytotoxicity profile is harmless.

### 3.1. Chemical Profile

The chemical profile of EtOAcAP and EtOAcR was previously characterized [26,32]. Ultra-high-performance liquid chromatography with high-resolution mass spectrometry (UHPLC–HRMS) demonstrated that gallic and ellagic acid and their derivatives, methylellagic acid O-hexoside, hydroxybenzoic and hydroxycinnamic acids, acylquinic acids, phenylethanoid glycosides, and flavonoids dominated in the EtOAcAP extract [32]. Seven compounds were isolated from the EtOAcR extract (cathechin, tormentic acid, niga-ichigoside F1, gein, 3,3′-di-O-methylellagic acid-4-O-β-d-glucopyranoside, 3-O-methylellagic acid-3′-O-α-3′′-O-acetylrhamnopyranoside and 3-O-methylellagic acid-3′-O-α-2′′-O-acetylrhamnopyranoside) and their structures were evaluated by comparison with their spectral characteristics (^1^H and ^13^C NMR, MS) [26].

### 3.2. Antimicrobial and Antioxidant Activities

Previously, the minimum inhibitory and bactericidal concentrations of the EtOAcR and EtOAcAP extracts against four *Staphylococcus* sp. (*S. aureus* NBIMCC 3359, ATCC 6538 P, NBIMCC 8327 and *S. epidermidis* NBIMCC 1093) were determined. The EtOAcAP extract showed stronger antistaphylococcal activity with MICs ranging between 39 and 313 μg/mL, compared to the EtOAcR extract with MICs between 156 μg/mL to 1.25 mg/mL. Against Gram-negative bacteria, the extracts showed no activity at the tested concentrations (the highest concentration was 2.5 mg/mL) [26]. However, in our previous studies, the inhibition of the phenotypic manifestations of the *Pseudomonas aeruginosa* Las/RhI quorum sensing system, such as biofilm formation, swarming motility, and pyocyanin synthesis by EtOAc extracts has been demonstrated [27,28]. The EtOAcR extract inhibited the *P. aeruginosa* ATCC 27853 biofilm formation by 83.8 ± 0.03% at 3.12 mg/mL (^1^/_4_MIC), compared to the EtOAcAP extract, which inhibited it by 61.5 ± 0.02% at 6.25 mg/mL (^1^/_4_MIC). At the same concentrations, both extracts slightly reduced the amount of pyocyanin synthesis. At ¼MIC, the EtOAcAP extract completely inhibited swarming motility (0 mm), compared to the EtOAcR extract (17.7 mm) [28]. At ¼MIC (1.56 mg/mL), the EtOAcR extract showed a higher effect against the PA01 swarming motility than EtOAcAP extract. At 1.25 mg/mL, both extracts decreased the PA01 pyocyanin synthesis fourfold [27]. In addition, the antiviral effect of both extracts was studied. The EtOAcAP extract was active against Coxsackie B type 1 and Human adenovirus type 5 (HAdV-C5), but the SI was three times higher for the EtOAcR to HAdV-C5 [32].

To clarify the antimicrobial effects of the two extracts, several additional analyses regarding antioxidant and redox-modulating activity were performed. The EtOAcAP extract contained a higher amount of total flavonoids (0.98 ± 0.05 μg quercetin/mg extract) and polyphenols (5.92 ± 0.23 μg gallic acid/mg extract), compared to the EtOAcR extract (0.22 ± 0.08 μg quercetin/mg extract and 0.96 ± 0.08 μg gallic acid/mg extract, respectively) [28]. Phenolic compounds play a key role in the antioxidant defense of the macroorganism by scavenging free radicals that damage deoxyribonucleic acid (DNA), proteins, lipids, and carbohydrates. In addition, they have been shown in many scientific studies to have the ability to donate electrons and hydrogen atoms, as well as to chelate metal cations [36]. According to the above and the results from the DPPH test, EtOAcAP exhibited three times higher activity than EtOAcR (19.50 ± 1.02 and 67.4 ± 3.21 mg/mL, respectively). The potential of EtOAcAP to scavenge the superoxide anion radical was four times greater than that of the EtOAcR extract (4.95 ± 0.048 and 17.84 ± 0.15 mg/mL, respectively). As iron and copper reduced, the EtOAcAP extract proved much stronger than EtOAcR [28].

### 3.3. Slime Production and Biofilm Formation of MRSA

Infections caused by MRSA include skin and soft tissue infections, bone and joint infections, pneumonia, bacteremia, and endocarditis. MRSA can be a part of the normal body flora, particularly in the nose [37]. After colonization, the viability of bacteria becomes a crucial pathogenic factor during infection, as small numbers of bacteria can be easily eliminated by the host defense system [38]. However, it can cause infections, especially in individuals with prolonged hospital stays, underlying health conditions, or following antibiotic use [37]. With the increased frequency of catheter utilization and implantation of medical devices and prostheses, the skin commensal staphylococci in particular have emerged as the most common group of pathogens implicated in human biofilm infections [39]. Some strains of *S. aureus* produce a slime, an extracellular polysaccharide layer that serves as a virulence factor. This slime promotes adhesion and protects the bacteria, which may partly explain the frequent therapeutic failures and the chronic nature of the infection [40]. On the other hand, not all strains of staphylococci can produce slime [40]. For this reason, our study began by examining the slime-forming potential of MRSA NBIMCC 8327 on the CRA plate (Figure 1). MRSA had a black center and a white periphery under the microscope, which proved its ability to form slime and therefore form a biofilm. Our research continued to determine the antibiofilm potential of EtOAcAP and EtOAcR extracts at sub-MICs against MRSA (Figure 2). The EtOAcAP extract inhibited biofilm formation in a dose-dependent manner, from 72.45 to 90.51%. Queve et al. [41] showed that biofilm formation by *S. aureus* was reduced after the application of a butanol extract fraction of *Rubus ulmifolius* Schott. (*Rosaceae*), in concentrations ranging from 50 to 200 µg/mL, which was rich in ellagic acid and its derivatives. On the other hand, gallic acid negatively affects the MRSA tolerance to low osmotic pressure and high salt concentration [42].

### 3.4. Gene Expression

The process of biofilm formation using *S. aureus* is not well studied, and only a few studies have been conducted related to the expression of genes involved in biofilm production. There are 12 genes responsible for biofilm formation (*fib*—fibrinogen-binding protein gene; *fnb*A and *fnb*B—fibronectin-binding protein genes; *ica*A, *ica*B, *ica*C, and *ica*D—adherence and intercellular adhesion genes; *clf*A and *clf*B—clumping factor genes; *ebps*—elastin-binding protein gene; *eno*—laminin-binding protein gene; and *cna*—collagen-binding protein gene) [43]. Most *S. aureus* strains form biofilm through PIA synthesis, which is encoded by conserved icaABCD operon [44,45]. The role of *ica*A and *ica*D genes is essential in PIA production [12], slime formation [46,47], and in the intercellular adhesion of the bacterial multilayer in the biofilm [47]. Therefore, the effect of EtOAc extracts at sub-MICs on the expression of these two genes was observed. According to the literature data, there are strains of *S. aureus* that form biofilms using proteins and extracellular DNA rather than polysaccharides (ica-independent biofilm formation) [48,49,50]. This process is controlled by various surface proteins, including Bap, clumping factors (ClfB), FnBPs, SasC, SasG, and protein A [51], as well as the regulatory proteins, such as Sar and Agr [52]. According to the literature data, the glucose-induced MRSA biofilms are *ica*-independent and involve protein instead of PIA/PNAG [50,53]. Our study shows biofilm inhibition in an *ica*-independent manner (Figure 3). The results obtained by RT-PCR showed that EtOAc extracts increased the *ica*A and *ica*D gene expression. Therefore, we assumed that the EtOAc extracts affect the fibronectin-binding proteins or other matrix components. The antibiofilm activity of *Vetiveria zizanioides* root extract was studied and downregulation was demonstrated in the *fnb*A, *fnb*B, and *clf*A genes involved in adhesins; there was no significant difference in the expression of *ica*A and *ica*D genes, which are responsible for biofilm maturation [54]. The QS *agr* system in *S. aureus* also controlled the maturation [55], and, therefore, future studies are needed to study the mechanism of action of the EtOAc extract. On the other hand, some authors showed that the upregulation of the *ica*A, and probably the *ica*D gene, can be explained as time dependent. At the 6th hour after treatment with the *Ginkgo biloba* L. exocarp extract, the relative expression of *ica*A increased three-fold compared to the control, and began to be downregulated after 8 h. The expression of the opposite of the icaABCD locus icaR was downregulated during the first 16 h after treatment with *G. biloba* exocarp extract and upregulated by 24 h [38]. This may be the second reason in our case, and at a later stage, there may be a decrease in *ica*A and *ica*D gene expression.

### 3.5. Cytotoxicity and Antiproliferative Activity

In patients with cancer, the functions of the anatomical barriers of the skin, such as desquamation, sweat pH, and normal flora of the skin, are often compromised by the uncontrollable growth of skin cells, which can form malignant tumors. For example, the risk of skin and soft tissue infections, including bacteremia, caused by *S. aureus* or coagulase-negative staphylococci, is increased in primary or metastatic skin tumors [56]. Therefore, in our previous study, the antineoplastic effect of both extracts on bladder cancer (T-24 and BC-3C) and liver carcinoma (HEP-G2) cells, as well as their cytotoxicity on normal embryonic kidney (HEK-293) cell lines, was investigated. The EtOAcAP showed the strongest antitumor activity on bladder cancer cell lines (IC_50_ = 21.33–25.28 µg/mL), compared to the EtOAcR extract, by inducing apoptosis and inhibiting NFkB p65 [32]. In this study, the potential of the two EtOAc extracts for antiproliferative activity on tumor skin cells was evaluated (Figure 4). The EtOAcAP extract was characterized by a higher SI for both tumorigenic cell lines A-375 (IC_50_ = 14.68 µg/mL) and A-431 (IC_50_ = 6.07 µg/mL) (Table 1). The EtOAcAP extract of aerial parts showed higher cytotoxicity against the HaCaT cell line (IC_50_ = 93 µg/mL, Figure 5) compared to CCL-1 cells (IC_50_ = 184.3 µg/mL, Table 1). Keratinocytes (HaCaT cell line) are located in the outermost layer of the skin and contribute to its strength and resistance, i.e., they perform a protective function [57]. Fibroblasts (CCL-1 cell line) are located in the connective tissue under the epithelium. They are mainly involved in the production and secretion of the extracellular matrix, containing collagen, elastin, and glycoproteins; in the dermis of the skin, they contribute to its strength and elasticity [58]. That is why keratinocytes are much more resistant than fibroblasts, due to their higher levels of antioxidant defenses and better DNA repair mechanisms [58,59]. Based on these results, the EtOAcAP extract was chosen to assess clonogenicity and the induction of apoptosis. The A-431 cell line showed strong sensitivity to the tested extract, as at a concentration of 7.5 µg/mL the clonogenicity was inhibited by over 90%, while A-375 cells were inhibited by approximately 55% (Figure 6). It is important to note that both cell lines differ in their origin. The A-431 cells were isolated from patients with squamous cell carcinoma (SCC), which develops in squamous cells in the outer layer of the skin. The A-375 cell line originated from a patient with melanoma, which develops in the cells (melanocytes) that produce melanin and is considered more aggressive. The number of melanoma cases worldwide is expected to increase from 325,000 and 57,000 deaths in 2020 to 510,000 and 96,000 deaths by 2040 [60]. At 7.5 µg/mL, the EtOAcAP extract induced apoptosis in the tested cells, compared to the nontumorogenic HaCaT cell line (Figure 7 and Figure 8). This concentration correlates excellently with the active concentrations against MRSA biofilm formation of EtOAcAP extract (4.88 and 9.77 µg/mL), which inhibit it by 72.45–86.37% (Figure 2). The IC_50_ value of the Iranian endemic plant *Teucrium persicum* Boiss. methanolic extract was 13 μg/mL in A-375 cell line [61], which is close to our results (Table 1). The cytotoxic activities of *Usnea aurantiaco-atra* (Jacq) Bory hexane and dichloromethane extracts against A-375 melanome cells were characterized by IC_50_ of 5.73 and 27.01 µg/mL [62]. The purified fraction of *Persicaria capitata* flowers showed IC_50_ = 475.22 µg/mL against SCC cells (A-431) [63]. The IC_50_ value of the hydro-alcoholic extract of *Manilkara zapota* extract was found to be 346.5 µg/mL [64]. According to these results, we believe that EtOAc extracts, especially those from the aerial parts, show promising results against melanoma and SCC.

### 3.6. In Vivo Experiments

The acute and subacute toxicity in a mouse model of the EtOAcAP extract after oral administration at 210, 70, and 20 mg/kg were previously examined. These concentrations were chosen according to the results of antimicrobial and antineoplastic activity of the EtOAcAP extract. The non-toxicity of the EtOAcAP extract was proven by the results of the histopathological analysis [32,65]. But *S. aureus* infections often lead to skin and soft tissue infections [66], especially in patients with SCC [67] and melanoma [68]. To ensure the safety of the EtOAcAP extract and the possibility of its application, a skin irritation test was performed at two concentrations—MIC = 313 µg/mL and 10xMIC = 3.13 mg/mL (Figure 9). The tested extract did not show any reaction, such as erythema and edema, making it safe for application to the skin against staphylococcal infections, especially in patients with SCC or melanoma.

## 4. Materials and Methods

### 4.1. Plant Material and Extraction

The dried plant materials from roots and aerial parts of *G. urbanum* were purchased from Sunny-Yambol, Ltd.^®^ (Yambol, Bulgaria) in April 2014 [26]. The extraction was performed as soon as possible after the herb arrived at the laboratory. Briefly, dried roots (500 g) and dried aerial parts (500 g) were macerated separately for 48 h in a total of 3 L methanol. The obtained solutions were filtered and concentrated by a vacuum rotary evaporator. Extraction was performed with solvents of increasing polarity: petroleum ether, EtOAc, and n-butanol. The extracts were evaporated to dry mass (10.4 g EtOAcAP extract and 14.1 g EtOAcR extract).

All experiments were performed in triplicate with fresh solutions of vacuum rotary dried extracts in DMSO. The toxic dose of DMSO is determined in advance and taken into account so that the solvent does not have an antibacterial and cytotoxic effect.

### 4.2. Antistaphylococcal Activity

#### 4.2.1. Bacterial Strain and Growth Condition

MRSA NBIMCC 8327 was cultured aerobically in brain heart infusion agar and broth (BHIA, M211 and BHIB, M210, HiMedia Laboratories Pvt. Ltd., Maharashtra, India) for 24 h at 37 °C.

#### 4.2.2. CRA Plate Test

The CRA method was used to check MRSA’s ability to produce slime and to form biofilm according to the protocol of Liberto et al. (2009) [18]. For this purpose, it was prepared as a medium with BHIA, 0.8% Congo red (573-58-0, Merck KGaA, Darmstadt, Germany), and 36 g of saccharose (Merck). The plate was incubated for 24 h at 37 °C. Slime-forming bacteria grow as black colonies, while those that do not produce slime grow as red colonies [18]. For reliability, the result was documented under a 25× microscope loupe.

#### 4.2.3. Determination to Inhibit MRSA Biofilm Formation

To evaluate the prevention of MRSA biofilm formation after treatment with sub-MICs of EtOAcR and EtOAcAP extracts, the previously described protocols were used [69,70]. MICs were previously determined (313 µg/mL for EtOAcR and 39 µg/mL for EtOAcAP extracts) [26]. A two-fold serial dilution was prepared in 96-well flat-bottom polystyrene plates containing BHIB broth supplemented with 0.5% glucose, with an initial concentration of the tested extracts of 625 µg/mL in a final volume of 100 µL per well. Then, an equivalent amount of bacterial culture at a concentration of 10^5^ CFU/mL was added. For sub-MICs, we choose the probes with final concentrations ranging from 39.1 to 156.25 µg/mL for EtOAcR and from 4.9 to 19.5 µg/mL for EtOAcAP extracts. The medium with bacteria is used as a positive control and the pure medium (blank) is used as negative control. The plates were incubated at 37 °C for 24 h, after which we followed the above-mentioned protocol [70] to determine the inhibition of MRSA biofilm formation.

#### 4.2.4. Isolation of RNA and cDNA Synthesis

The Environmental DNA & RNA Purification Kit (E3572, EURx Ltd., Gdańsk, Poland) was used to isolate RNA from treated probes with sub-MICs of extracts (4.9 µg/mL and 19.5 µg/mL for EtOAcAP, and 39 µg/mL and 156 µg/mL for EtOAcR) and untreated positive MRSA control. The protocol of the manufacturer was followed with some optimization. Fifty µL lysozyme (20 mg/mL) from chicken egg white (L6876, Merck Sigma-Aldrich, Saint Louis, MI, USA) and 10 µL lysostaphin (L4402, Merck Sigma-Aldrich, Saint Louis, MI, USA) from *Staphylococcus staphylolyticus* (200 U/mL) were added per probe. Concentrations of RNA were measured on a Thermo Scientific NanoDrop Lite Spectrophotometer (Temecula, CA, USA) and standardized to equal concentrations of RNA (1 μg in 20 μL) according to the protocol of iScript Select cDNA Synthesis Kit (Bio-Rad Laboratories, Inc., 170-8896, Hercules, CA, USA) for reverse-transcription PCR (RT-PCR) on C1000 Touch^TM^ PCR Thermal Cycler (Bio-Rad Laboratories, Inc., Singapore, Southeast Asia). The probes were subjected to cDNA synthesis using random hexamer primers and reverse transcriptase enzymes. The obtained cDNAs were stored at −80 °C and used for real-time RT-PCR.

#### 4.2.5. Real-Time PCR

To determine the expression of the *ica*A and *ica*D genes after normalization with the reference *rho* gene (Table 2), quantitative real-time PCR was prepared using Sso Advanced Universal SYBR Green Supermix (Bio-Rad Bio-Rad Laboratories, Inc., 1725270, Hercules, CA, USA) on a CFX96 Touch^TM^ Real-Time Detection System (Bio-Rad, Singapore, Southeast Asia).

#### 4.2.6. Gene Expression Analysis

The effect of the tested extracts at sub-MICs on *ica*A and *ica*D gene expression levels in MRSA was determined by the 2^−∆∆Ct^ method. For the normalization of the data, *rho* was used as a housekeeping gene.

#### 4.2.7. Cell Line and Culture Conditions

Three cell lines were used in this study. They include A-375 (malignant melanoma), A-473 (epidermoid skin carcinoma) and HaCaT (normal keratinocytes) originating from the biobank for Cell Cultures Cytion GmbH, Eppelheim, Germany. The cells were cultured under standard conditions (37 °C, 5% CO2, maximum humidity). The experiments were performed under sterile conditions in a laminar flow cabinet (Telstar Bio II Advance, Terrassa, Spain) with protection class II. The cells were maintained in DMEM culture medium (DMEM-HA, Capricorn Scientific GmbH, Ebsdorfergrund, Germany) enriched with 2 mM L-glutamine (STA-B, Capricorn Scientific GmbH, Germany) and 10% fetal calf serum (FBS-HI-11B, Capricorn Scientific GmbH, Germany). To prevent microbial contamination, a solution of penicillin (PEN)/streptomycin (S) (PS-B, Capricorn Scientific GmbH, Germany) was added to the medium to a final concentration of 100,000 Units/L PEN and 100 mg/L S. Cells were sub-cultured twice a week by being diluted at a ratio of 1:8, as per the recommendations of the biobank.

#### 4.2.8. MTT Assay

This method was used to determine the mean inhibitory concentrations (IC50) of the tested extract. The protocol in Annex C of the international standard ISO 10993-5:2009 [73] was applied, based on the Mossman method with some modifications [73,74,75,76,77,78]. The cell lines were treated with the extract during the exponential growth phase (so-called log phase), when the fraction of dividing cells was high (usually above 90%). Briefly, the cells were plated in 96-well plates in a volume of 100 µL 24 h before treatment with both extracts at concentrations ranging from 0.001 to 0.5 mg/mL in two-fold serial dilutions. A minimum of 4 replicates were made for each sample. The cytotoxic effect was recorded at the 72nd h from the start of the treatment. For this purpose, the tetrazole salt MTT (M2128-1G, Sigma-Aldrich, Steinheim, Germany) was added to the samples in a concentration of 0.05 mg/mL, and they were incubated for 120 min at 37 °C. The products formed in the living cells, formazan crystals, were dissolved with an equivalent amount of organic solvent (5% HCOOH in 2-propanol), and the absorption was measured on an ELISA microreader ELx800 (BioTek, Shoreline, WA, USA) at a wavelength of λ = 550 nm and the reference filter at λ = 690 nm. The absorption of the pure solvent was measured in parallel as blank sample, and its value was subtracted from that of the samples.

#### 4.2.9. Colony Forming Unit Assay

The death of the treated cells can occur over a period varying from several hours to several days. Measuring the ability of surviving cells to form colonies reveals their potential further proliferation. This method became the gold standard for assessing cell sensitivity and determining the percentage of vital versus apoptotic cell fractions in experimental conditions. Cells were treated with the extract at concentrations of EtOAcAP 3.75, 7.5 and 15 µg/mL, then plated on semi-solid methylcellulose medium and cultured at 37 °C and 5% CO_2_ for 10 days. The number of colonies in the samples was calculated as a percentage of the untreated control, conditionally accepted as 100%. The standard deviation for each applied concentration, as well as for the untreated control, was also calculated in percentages.

#### 4.2.10. Determination of Caspase 3 Activity

For this purpose, a Biotium kit (Caspase-3 DEVD-R110 Fluorimetric&colorimetric Assay Kit, Fremont, CA, USA), developed for colorimetric measurement of the activity of the caspase 3 enzyme at λ = 495 nm, was used. Briefly, A-375 and A-431 cells were seeded at the same density as for the MTT assay, but in 5 mL volumes in tissue-treated Petri dishes for cultivating adherent cell cultures. After 24 h of incubation at 37 °C and 5% CO_2_, the samples were treated with the following concentrations of the EAS extract: 0, 3.75, 7.5, and 15 µg/mL (A-375) and 0, 3.75, 7.5, 15, and 30 µg/mL (A-431). After 48 h of incubation, the samples were processed according to the manufacturer’s instructions for the test used, and the result was measured spectrophotometrically on an ELISA reader BioTek ELx800 at the above-mentioned wavelength.

#### 4.2.11. Hoechst Staining

Hoechst 33342 (14533, Sigma^®^ Life Science/Merck KGaA, Darmstadt, Germany) was used for staining DNA fragmentation in live cells (HaCaT, A-375 and A-431). Briefly, cells were plated as described for the caspase 3 activity assay, and treated with concentrations 7.5, 15, and 30 µg/mL for EtOAcAP for HaCaT and A-375 cells, and concentrations 3.75, 7.5, and 15 µg/mL EtOAcAP for A-431 cells. After 48 h of incubation, the cells in the samples were washed three times with PBS. Hoechst solution in PBS (100 µg/mL final concentration) was added for 20 min [79] and the micrographs were taken under a Nikon Eclipse Ti-U CLSM (20 × plan apochromatic objective, microscopic magnification 200×). The image acquisition was performed with the EZ-C1 software (version 2.2) of the microscope.

#### 4.2.12. Statistical Methods

All experiments were performed in triplicate and presented as average values ± standard deviation (SD) using ANOVA and GraphPad Prizm software (Version 6.0.0 for Windows, GraphPad Software, Boston, MA, USA). A *p*-value < 0.05 was accepted as statistically significant. The medium inhibitory concentrations were calculated with a mathematical model for nonlinear regression in the GraphPad Prizm software. The same software was used for analyzing the data from the CFU and caspase 3 assays. Two-way ANOVA (Tukey’s multiple comparisons test) was performed for statistical analysis of the different groups of treated cells.

#### 4.2.13. Skin Irritation Test

The potential of EtOAcAP and EtOAcR extracts to cause dermal irritation was assessed according to ISO 10993-10 [80], as described before [81]. A healthy young albino rabbit with intact skin was used, which was acclimatized according to ISO 10993-2 [82] and Rule No. 20 of 1.11.2012 [83]. Briefly, the fur on the back of the rabbit was clipped (10 × 15 cm) with Artero Premium 2 Speed (Ref. M339, Singapore, Southeast Asia) 4 h before the test. A 0.5 mL amount of the test extract with a favorable cytotoxicity profile and high antibiofilm activity, as well as controls (negative—USP water and positive—10% Sodium dodecyl sulfate (SDS, L5750, Merck Sigma-Aldrich, Saint Louis, MI, USA)), were applied directly to the skin and covered with an absorbent gauze pad. A semi-occlusive dressing was applied to the application site for 4 h. The test areas were marked with a permanent marker. The treated areas were carefully rinsed with lukewarm water and the skin was carefully dried. After removal of the non-occlusive dressings, reactions at each application site were observed and scored for erythema and/or edema according to the scoring system given in ISO 10993-10 at the 1st, 24th, 48th, and 72nd hour. The primary irritation index (PII) was calculated based on the primary irritation score (PIS) for each sample, and the results were read based on the scoring system for skin reaction.

## 5. Conclusions

The conducted study on the antistaphylococcal and antineoplastic activity of both EtOAc extracts showed that the EtOAcAP extract exhibits a much higher activity compared to the EtOAcR extract, and it reveals strong potential for further pharmacological development regarding skin application. This might be due to the higher content of phenolic compounds. The EtOAcAP extract inhibited the MRSA growth at 39 µg/mL and reduced the biofilm formation with 72.4–90.5% at sub-MICs varying between 4.88 and 19.53 µg/mL. Induction in the expression of the *ica*A and *ica*D genes was observed by real-time PCR, possibly because MRSA uses surface proteins to form a biofilm in an *ica*-independent manner or because after exposure to the extract the bacterial cells are in a “shock” condition. The same extract demonstrated strong antineoplastic activity with IC_50_ = 6.07–14.68 µg/mL against two tumorigenic cell lines derived from patients with SCC (A-431) and melanoma (A-375) by inducing apoptosis at 3.75 and 7.5 µg/mL. The EtOAcAP extract did not show any skin irritation reaction, which makes it promising for local application in the adjuvant therapy of cutaneous malignant diseases where MRSA infections play a key role in processes of skin inflammation and the progression of carcinogenesis.

## Figures and Tables

**Table 1:** Median inhibitory concentrations of EtOAc extracts from *G. urbanum*.

Cell Line	Extract	Parameters			
		IC_50_ (µg/mL)	CI 95%	R^2^	SI
A-375	EtOAcAP	14.68	13.60 to 15.83	0.99	12.55
	EtOAcR	23.67	22.20 to 25.24	0.99	4.08
A-431	EtOAcAP	6.07	5.24 to 7.03	0.97	30.36
	EtOAcR	8.71	7.86 to 9.65	0.98	11.11
CCL-1	EtOAcAP	184.3	164.1 to 206.9	0.96	-
	EtOAcR	96.73	86.84 to 107.7	0.97	-

**Table 2:** List of the primers, their sequences, and melting temperatures (Tm) used in this investigation.

Primers	Sequences	Tm	Reference
*ica*A F	ACACTTGCTGGCGCAGTCAA	69.4 °C	[71]
*ica*A R	TCTGGAACCAACATCCAACA	64.1 °C	
*ica*D F	ATGGTCAAGCCCAGACAGAG	64.3 °C	
*ica*D R	AGTATTTTCAATGTTTAAAGCAA	56.4 °C	
*rho* F	GGAAGATACGACGTTCAGAC	58.8	
*rho* R	GAAGCGGGTGGAAGTTTA	60.3	[72]

## Data Availability

The original contributions presented in this study are included in the article/Supplementary Material. Further inquiries can be directed to the corresponding author.

## Supplementary Materials

The following supporting information can be downloaded at: https://www.mdpi.com/article/10.3390/antibiotics14070627/s1, Table S1. Two-way ANOVA analysis of the biofilm formation data. Table S2. Two-way ANOVA analysis of the relative gene expression of *ica*A gene. Table S3.Two-way ANOVA analysis of the relative gene expression of *ica*D gene. Table S4. Two-way ANOVA for statistical evaluation of the data from the CFU assay. Table S5. Two-way ANOVA analysis of the data from the caspase3 activity assay.
