# Supplementary material for: Inhibition of the MRSA Biofilm Formation and Skin Antineoplastic Activity of Ethyl Acetate Roots and Aerial Parts Extracts from Geum urbanum L
Source: Antibiotics (Basel). 2025 Jun 20;14(7):627. doi: 10.3390/antibiotics14070627 (PMC12291766; doi:10.3390/antibiotics14070627)
Supplement: Supplementary file 1 [file antibiotics-14-00627-s001.zip › antibiotics-3702739-supplementary.pdf]

Table S1. Two-way ANOVA analysis of the biofilm formation data.

Tukey's multiple comparisons test	Mean Diff.	95.00% CI of diff.	Summary	Adjusted P Value
0:EtOAcAP vs. 0:EtOAcR	0.000	-0.2886 to 0.2886	ns	>0.9999
0:EtOAcAP vs. 1/2 MIC:EtOAcAP	2.768	2.479 to 3.056	****	<0.0001
0:EtOAcAP vs. 1/2 MIC:EtOAcR	0.5612	0.2726 to 0.8498	***	0.0001
0:EtOAcAP vs. 1/4 MIC:EtOAcAP	2.637	2.349 to 2.926	****	<0.0001
0:EtOAcAP vs. 1/4 MIC:EtOAcR	0.5576	0.2690 to 0.8462	***	0.0001
0:EtOAcAP vs. 1/8 MIC:EtOAcAP	2.205	1.917 to 2.494	****	<0.0001
0:EtOAcAP vs. 1/8 MIC:EtOAcR	0.5116	0.2230 to 0.8002	***	0.0003
0:EtOAcR vs. 1/2 MIC:EtOAcAP	2.768	2.479 to 3.056	****	<0.0001
0:EtOAcR vs. 1/2 MIC:EtOAcR	0.5612	0.2726 to 0.8498	***	0.0001
0:EtOAcR vs. 1/4 MIC:EtOAcAP	2.637	2.349 to 2.926	****	<0.0001
0:EtOAcR vs. 1/4 MIC:EtOAcR	0.5576	0.2690 to 0.8462	***	0.0001
0:EtOAcR vs. 1/8 MIC:EtOAcAP	2.205	1.917 to 2.494	****	<0.0001
0:EtOAcR vs. 1/8 MIC:EtOAcR	0.5116	0.2230 to 0.8002	***	0.0003
1/2 MIC:EtOAcAP vs. 1/2 MIC:EtOAcR	-2.206	-2.495 to -1.918	****	<0.0001
1/2 MIC:EtOAcAP vs. 1/4 MIC:EtOAcAP	-0.1303	-0.4189 to 0.1583	ns	0.7639
1/2 MIC:EtOAcAP vs. 1/4 MIC:EtOAcR	-2.210	-2.499 to -1.921	****	<0.0001
1/2 MIC:EtOAcAP vs. 1/8 MIC:EtOAcAP	-0.5623	-0.8509 to -0.2737	***	0.0001
1/2 MIC:EtOAcAP vs. 1/8 MIC:EtOAcR	-2.256	-2.545 to -1.967	****	<0.0001
1/2 MIC:EtOAcR vs. 1/4 MIC:EtOAcAP	2.076	1.787 to 2.365	****	<0.0001

1/2 MIC:EtOAcR vs. 1/4 MIC:EtOAcR	-0.003667	-0.2923 to 0.2849	ns	>0.9999
1/2 MIC:EtOAcR vs. 1/8 MIC:EtOAcAP	1.644	1.355 to 1.933	****	<0.0001
1/2 MIC:EtOAcR vs. 1/8 MIC:EtOAcR	-0.04967	-0.3383 to 0.2389	ns	0.9984
1/4 MIC:EtOAcAP vs. 1/4 MIC:EtOAcR	-2.080	-2.368 to -1.791	****	<0.0001
1/4 MIC:EtOAcAP vs. 1/8 MIC:EtOAcAP	-0.4320	-0.7206 to -0.1434	**	0.0018
1/4 MIC:EtOAcAP vs. 1/8 MIC:EtOAcR	-2.126	-2.414 to -1.837	****	<0.0001
1/4 MIC:EtOAcR vs. 1/8 MIC:EtOAcAP	1.648	1.359 to 1.936	****	<0.0001
1/4 MIC:EtOAcR vs. 1/8 MIC:EtOAcR	-0.04600	-0.3346 to 0.2426	ns	0.9990
1/8 MIC:EtOAcAP vs. 1/8 MIC:EtOAcR	-1.694	-1.982 to -1.405	****	<0.0001

Table S2. Two-way ANOVA analysis of the relative gene expression of *ica*A gene.

Tukey's multiple comparisons test	Mean Diff.	95.00% CI of diff.	Summary	Adjusted P Value
Control:EtOAcAP vs. Control:EtOAcR	0.000	-0.1585 to 0.1585	ns	>0.9999
Control:EtOAcAP vs. 1/8 MIC:EtOAcAP	-0.3735	-0.5320 to -0.2150	****	<0.0001
Control:EtOAcAP vs. 1/8 MIC:EtOAcR	-0.1948	-0.3533 to -0.03631	*	0.0137
Control:EtOAcAP vs. 1/2 MIC:EtOAcAP	-0.2636	-0.4221 to -0.1051	**	0.0013

Control:EtOAcAP vs. 1/2 MIC:EtOAcR	-0.7034	-0.8619 to -0.5449	****	<0.0001
Control:EtOAcR vs. 1/8 MIC:EtOAcAP	-0.3735	-0.5320 to -0.2150	****	<0.0001
Control:EtOAcR vs. 1/8 MIC:EtOAcR	-0.1948	-0.3533 to -0.03631	*	0.0137
Control:EtOAcR vs. 1/2 MIC:EtOAcAP	-0.2636	-0.4221 to -0.1051	**	0.0013
Control:EtOAcR vs. 1/2 MIC:EtOAcR	-0.7034	-0.8619 to -0.5449	****	<0.0001
1/8 MIC:EtOAcAP vs. 1/8 MIC:EtOAcR	0.1787	0.02017 to 0.3372	*	0.0243
1/8 MIC:EtOAcAP vs. 1/2 MIC:EtOAcAP	0.1099	-0.04859 to 0.2684	ns	0.2546
1/8 MIC:EtOAcAP vs. 1/2 MIC:EtOAcR	-0.3299	-0.4884 to -0.1714	***	0.0002
1/8 MIC:EtOAcR vs. 1/2 MIC:EtOAcAP	-0.06876	-0.2273 to 0.08976	ns	0.6951
1/8 MIC:EtOAcR vs. 1/2 MIC:EtOAcR	-0.5086	-0.6671 to -0.3501	****	<0.0001
1/2 MIC:EtOAcAP vs. 1/2 MIC:EtOAcR	-0.4398	-0.5984 to -0.2813	****	<0.0001

Table S3.Two-way ANOVA analysis of the relative gene expression of *ica*D gene.

Tukey's multiple comparisons test	Mean Diff.	95.00% CI of diff.	Summary	Adjusted P Value
Control:EtOAcAP vs. Control:EtOAcR	0.000	-0.1585 to 0.1585	ns	>0.9999
Control:EtOAcAP vs. 1/8 MIC:EtOAcAP	-0.2821	-0.4406 to -0.1237	***	0.0007
Control:EtOAcAP vs. 1/8 MIC:EtOAcR	-0.2636	-0.4221 to -0.1051	**	0.0013
Control:EtOAcAP vs. 1/2 MIC:EtOAcAP	-0.6555	-0.8140 to -0.4971	****	<0.0001
Control:EtOAcAP vs. 1/2 MIC:EtOAcR	-0.02067	-0.1792 to 0.1378	ns	0.9974
Control:EtOAcR vs. 1/8 MIC:EtOAcAP	-0.2821	-0.4406 to -0.1237	***	0.0007
Control:EtOAcR vs. 1/8 MIC:EtOAcR	-0.2636	-0.4221 to -0.1051	**	0.0013
Control:EtOAcR vs. 1/2 MIC:EtOAcAP	-0.6555	-0.8140 to -0.4971	****	<0.0001
Control:EtOAcR vs. 1/2 MIC:EtOAcR	-0.02067	-0.1792 to 0.1378	ns	0.9974
1/8 MIC:EtOAcAP vs. 1/8 MIC:EtOAcR	0.01856	-0.1399 to 0.1770	ns	0.9985
1/8 MIC:EtOAcAP vs. 1/2 MIC:EtOAcAP	-0.3734	-0.5319 to -0.2149	****	<0.0001
1/8 MIC:EtOAcAP vs. 1/2 MIC:EtOAcR	0.2615	0.1030 to 0.4200	**	0.0014
1/8 MIC:EtOAcR vs. 1/2 MIC:EtOAcAP	-0.3920	-0.5504 to -0.2335	****	<0.0001
1/8 MIC:EtOAcR vs. 1/2 MIC:EtOAcR	0.2429	0.08443 to 0.4014	**	0.0025
1/2 MIC:EtOAcAP vs. 1/2	0.6349	0.4764 to 0.7934	****	<0.0001

MIC:EtOAcR				

Table S4. Two-way ANOVA for statistical evaluation of the data from the CFU assay.

**Tukey's multiple comparisons test**	**Mean Diff.**	**95% CI of diff.**	**Summary**	**Adjusted P Value**
0:A-375 vs. 0:A-431	60.67	15.05 to 106.3	**	0.0058
0:A-375 vs. 3.75:A-375	-1.667	-47.28 to 43.95	ns	> 0.9999
0:A-375 vs. 3.75:A-431	33.00	-12.62 to 78.62	ns	0.2556
0:A-375 vs. 7.5:A-375	201.3	155.7 to 246.9	****	< 0.0001
0:A-375 vs. 7.5:A-431	333.7	288.1 to 379.3	****	< 0.0001
0:A-375 vs. 15:A-375	206.2	155.2 to 257.2	****	< 0.0001
0:A-375 vs. 15:A-431	365.7	320.1 to 411.3	****	< 0.0001
0:A-431 vs. 3.75:A-375	-62.33	-107.9 to -16.72	**	0.0046
0:A-431 vs. 3.75:A-431	-27.67	-73.28 to 17.95	ns	0.4460
0:A-431 vs. 7.5:A-375	140.7	95.05 to 186.3	****	< 0.0001
0:A-431 vs. 7.5:A-431	273.0	227.4 to 318.6	****	< 0.0001
0:A-431 vs. 15:A-375	145.5	94.50 to 196.5	****	< 0.0001
0:A-431 vs. 15:A-431	305.0	259.4 to 350.6	****	< 0.0001
3.75:A-375 vs. 3.75:A-431	34.67	-10.95 to 80.28	ns	0.2104
3.75:A-375 vs. 7.5:A-375	203.0	157.4 to 248.6	****	< 0.0001
3.75:A-375 vs. 7.5:A-431	335.3	289.7 to 380.9	****	< 0.0001
3.75:A-375 vs. 15:A-375	207.8	156.8 to 258.8	****	< 0.0001
3.75:A-375 vs. 15:A-431	367.3	321.7 to 412.9	****	< 0.0001
3.75:A-431 vs. 7.5:A-375	168.3	122.7 to 213.9	****	< 0.0001
3.75:A-431 vs. 7.5:A-431	300.7	255.1 to 346.3	****	< 0.0001
3.75:A-431 vs. 15:A-375	173.2	122.2 to 224.2	****	< 0.0001

3.75:A-431 vs. 15:A-431	332.7	287.1 to 378.3	****	< 0.0001
7.5:A-375 vs. 7.5:A-431	132.3	86.72 to 177.9	****	< 0.0001
7.5:A-375 vs. 15:A-375	4.833	-46.17 to 55.83	ns	> 0.9999
7.5:A-375 vs. 15:A-431	164.3	118.7 to 209.9	****	< 0.0001
7.5:A-431 vs. 15:A-375	-127.5	-178.5 to -76.50	****	< 0.0001
7.5:A-431 vs. 15:A-431	32.00	-13.62 to 77.62	ns	0.2861
15:A-375 vs. 15:A-431	159.5	108.5 to 210.5	****	< 0.0001

Table S5. Two-way ANOVA analysis of the data from the caspase3 activity assay.

**Tukey's multiple comparisons test**	**Mean Diff.**	**95.00% CI of diff.**	**Summary**	**Adjusted P Value**
0:A-375 vs. 0:A-431	-0.08700	-0.1026 to -0.07138	****	<0.0001
0:A-375 vs. 3.75:A-431	-0.07800	-0.09362 to -0.06238	****	<0.0001
0:A-375 vs. 7.5:A-375	0.009000	-0.006624 to 0.02462	ns	0.5840
0:A-375 vs. 7.5:A-431	-0.2100	-0.2256 to -0.1944	****	<0.0001
0:A-375 vs. 15:A-375	-0.03900	-0.05462 to -0.02338	****	<0.0001
0:A-375 vs. 15:A-431	-0.2750	-0.2906 to -0.2594	****	<0.0001
0:A-375 vs. 30:A-375	-0.001000	-0.01662 to 0.01462	ns	>0.9999
0:A-431 vs. 3.75:A-431	0.009000	-0.006624 to 0.02462	ns	0.5840
0:A-431 vs. 7.5:A-375	0.09600	0.08038 to 0.1116	****	<0.0001
0:A-431 vs. 7.5:A-431	-0.1230	-0.1386 to -0.1074	****	<0.0001
0:A-431 vs. 15:A-375	0.04800	0.03238 to 0.06362	****	<0.0001
0:A-431 vs. 15:A-431	-0.1880	-0.2036 to -0.1724	****	<0.0001

0:A-431 vs. 30:A-375	0.08600	0.07038 to 0.1016	****	<0.0001
3.75:A-431 vs. 7.5:A-375	0.08700	0.07138 to 0.1026	****	<0.0001
3.75:A-431 vs. 7.5:A-431	-0.1320	-0.1476 to -0.1164	****	<0.0001
3.75:A-431 vs. 15:A-375	0.03900	0.02338 to 0.05462	****	<0.0001
3.75:A-431 vs. 15:A-431	-0.1970	-0.2126 to -0.1814	****	<0.0001
3.75:A-431 vs. 30:A-375	0.07700	0.06138 to 0.09262	****	<0.0001
7.5:A-375 vs. 7.5:A-431	-0.2190	-0.2346 to -0.2034	****	<0.0001
7.5:A-375 vs. 15:A-375	-0.04800	-0.06362 to -0.03238	****	<0.0001
7.5:A-375 vs. 15:A-431	-0.2840	-0.2996 to -0.2684	****	<0.0001
7.5:A-375 vs. 30:A-375	-0.01000	-0.02562 to 0.005624	ns	0.4484
7.5:A-431 vs. 15:A-375	0.1710	0.1554 to 0.1866	****	<0.0001
7.5:A-431 vs. 15:A-431	-0.06500	-0.08062 to -0.04938	****	<0.0001
7.5:A-431 vs. 30:A-375	0.2090	0.1934 to 0.2246	****	<0.0001
15:A-375 vs. 15:A-431	-0.2360	-0.2516 to -0.2204	****	<0.0001
15:A-375 vs. 30:A-375	0.03800	0.02238 to 0.05362	****	<0.0001
15:A-431 vs. 30:A-375	0.2740	0.2584 to 0.2896	****	<0.0001
